# Multifarious feed additives on lamb performance on Kuwait farms

**DOI:** 10.14202/vetworld.2022.2785-2794

**Published:** 2022-12-05

**Authors:** Hana’a Burezq, Faten Khalil

**Affiliations:** Desert Agriculture and Ecosystems Program, Environment and Life Sciences Research Center, Kuwait Institute for Scientific Research, Safat 13109, Kuwait

**Keywords:** ammonium chloride, efficiency, feed additives, fishmeal, performance, urea

## Abstract

**Background and Aim:**

A change in the livestock feeding strategy is of utmost importance for the stability of animal health and sustainable livestock productivity to overcome the problem of subsiding the environmental effects of sheep production. Supplementing dietary feed with safe and efficient additives provides optimal animal performance and maximizes productivity. This study aimed to assess the effects of adding various feed additives to lamb rations for optimizing feed efficiency in weaned lambs for meat production in Kuwait.

**Materials and Methods:**

The feed additives, namely, ammonium chloride, urea, algae, fishmeal, and humic acid, were investigated on the physical performance of lambs for their effect on body weight, length, height, and waist length. The total feed consumption rate and feed efficiency were also measured. Each treatment comprising five healthy lambs was randomly allocated into six treatments comprising 30 lambs. The six treatments were the basal ration supplemented with ammonium chloride (50–100 g/day/head), urea (30 g/day/head), fishmeal (35 g/day/head), algae (*Spirulina platensis*) powder (50 g/day/head), humic acid (2.5 g/day/head), control group with only basal ration. The study was conducted for around 27 months and the data were recorded once in 2 weeks.

**Results:**

The results indicated a positive elevation in the physique of lambs with all tested additives, showing an affirmative insignia for lamb fattening. The growth parameters in terms of augmented length, height, and waist length of lambs’ bodies amplified significantly with ammonium chloride and fishmeal supplement, while the other additives reported a non-significant increment. The feed consumption was significantly elevated for ammonium chloride, algae, and fishmeal supplementation, while humic acid was recorded the least. Concerning feed efficiency of young lambs, fish meal and ammonium chloride were reported best, followed by urea. In contrast, algae and humic acid exhibited a non-significant effect on feed efficiency.

**Conclusion:**

This study exposed noteworthy influence on a lamb body’s performance with the addition of fish meal and ammonium chloride in lamb rations, trailed by urea and algae.

## Introduction

The global livestock sector is highly dynamic and evolving in response to the rapid increase in demand for livestock byproducts driven by the increasing world population [1]. The vigorous growth in livestock production has made the production and management of livestock systems very difficult [2]. Nutrition is the key factor in enhancing sheep’s health and welfare [3]. Feed is an integral part of the animal food chain, playing a key role in growth, productivity, and welfare, and the composition, safety, and quality of their byproducts [4]. The most promising nutritional strategy to amplify feed efficiency and digestibility is the inclusion of materials that enrich feed quality [5]. Animal diet comprises materials of plant, animal, pharmaceutical, and industrial origin, developed to attain the objective of animal performance [6]. Higher concentrate mixtures are needed in animal diets, especially in lactating animals, for their healthy progeny [7, 8]. Although additives do not meet any nutritional livestock requirement, supplemented to the basic feed ration, boast growth and other productive functions of the animal body, increase feed efficiency and use, preserve feed, and other animal health benefits and metabolism [9, 10]. Feed additives improve livestock’s digestive and production efficiency with minimal ecosystem effects [11]. Nutritional feed additives used in animal nutrition, instigated from diverse sources, affect physiological processes, including nutrient digestibility and absorption, immunity, mineral status, antioxidant activity, or livestock reproduction. Improving nutrient digestion and absorption by feed supplementation can amplify micronutrient availability, provide safe and functional foods, and decrease environmental pollution from animal production [12]. Feed additives represent different molecules, compounds, or organisms that promote ingestion, absorption and assimilation of nutrients, growth, and health, and affect physiological processes, such as the immune system, stress resistance, and reproduction [13]. Feed additives are products that could be incorporated into the total feed formula in a very small proportion to improve the overall feed conversion efficiency [14]. Some feed additives have the ability to enhance rumen fermentation by increasing microbial activity, thereby digestibility of rations. These additives were found to stimulate microorganisms in the rumen and, in that way, increased feed efficiency and usage [15–18].

Feed additives are widely used worldwide for the welfare of animals. Several negative effects were noticed due to the inclusion of additives, especially antibiotics. Antibiotics inhibit absorption from the intestine by toxin formation, which has an adverse impact on animal health. The growth-promoting ability of antibiotics is hindered by toxins, by harmful organisms. After being fed over time, they retain the strains of bacteria resistant to antibiotics, which proliferate in animals, and are transmitted to other animals on interaction, forming colonies of antibiotic-resistant bacteria [19]. In European countries, they have regulated products that can be placed on the market only if they have been authorized for use and used only for the reason stated within the authorization. Additives that have been through an authorization procedure may only be placed on the market and used. Authorization is granted for feed intended for specific animal species or categories and specific conditions of use [20, 21]. The EU has already banned antibiotics used in human medicine from being added to animal feed. Monensin sodium, Salinomycin sodium, avilamycin, and flavophospholipol have been banned since 2006 in European countries. Hence, the right choice of feed additives matters in any animal experiment to ensure the safety of the test animals.

Kuwait accounts for 588,618 heads of sheep and 11%–12% of the red meat needs of the country are met by the sheep industry. The high nutritional value of lamb meat in Kuwait increases the interest in improving the local production yield of this animal species. About 30% of imported feeds were wasted due to the absence of proper ration preparation, storage, feeding methods, feed refusal by the animals, and use of nutrients [22].

To augment sheep production for meat purposes in Kuwait, an investigation was conducted in the Kuwait Institute for Scientific Research (KISR) with relatively safe assorted feed additives, including urea, ammonium chloride, fishmeal, algae, and humic acid on the physical performance (body weight, body length [BL], body height, and waist length) and efficiency in feed consumption.

## Materials and Methods

### Ethical approval

This study was conducted under the project FA150C, which was ethically approved by KISR, Kuwait.

### Study period and location

The study was conducted from October 2019 to September 2021 at Livestock Research Centre, Suleibiya, belonging to KISR and Public Authority for Agriculture and Fish Resources.

### Experimental animals

Thirty healthy lambs (23.33 ± 1.06 kg body weight and 4–4.5 months old lambs as it is the weaning age) were randomly allocated into six treatments [23], each treatment comprising five lambs.

- T1: Basal ration supplemented with ammonium chloride (50–100 g/day/head).
- T2: Basal ration supplemented with urea (30 g/day/head).
- T3: Basal ration supplemented with fishmeal (35 g/day/head).
- T4: Basal ration supplemented with algae (*Spirulina platensis*) powder (50 g/day/head).
- T5: Basal ration supplemented with humic acid (2.5 g/day/head).
- T6: Control group with basal ration.

The basal ration comprised roughage, concentrates, vitamins, and minerals, as depicted in Table-1. The basal ration was developed to meet the lamb’s nutrient requirements to balance the body weight gain at a rate of 0.3 kg/day [23]. Rations with a C: R ratio of 80C:20R were used as per KISR’s feeding and a previous nutritional study [24]. The basic ration provides the animals with phosphorus (5.00%), calcium (18.00%), sodium (5.00%), magnesium (5.00%), manganese (500 mg/kg (as manganese oxide); cobalt (100 mg/kg (as cobaltous sulfate), zinc (2000 mg/kg) (as zinc oxide); iodine (125 mg/kg (as calcium iodide); selenium (10 mg/kg (as sodium selenite); vitamin A (400,000 IU/kg; vitamin D3 (100,000 IU/kg); and vitamin E (Alpha Tocopherol). The other feed additives used in the experiment (ammonium chloride, urea, fish meal, and algae) were procured from cooperatives. The feed was offered twice daily in the morning and evening with access to water. The trial period was 26 weeks, with a pre-trial period of 1 week for adaptation to diets and facilities. Animals were weighed every 2 weeks, and the total number of readings (recording data) was thirteen periods. The feed comprised roughage, concentrates, vitamins, and minerals, as shown in Table-1.

**Table-1:** Ingredients of basic rations on dry basis, vitamin, and mineral composition.

Ingredients	70C: 30R (Used for ewes and rams)	80C: 20R (Used for young lambs)
A. Concentrates		
Barley	40.5	51.0
Wheat bran	10.0	10.0
Corn	10.0	10.0
Soybean meal	6.5	6.0
*Vitamins and minerals	1.0	1.0
Limestone	1.0	1.0
Salt	1.0	1.0
Total A	70.0	80.0
B. Roughages		
Alfalfa Hay	15.0	10.0
Wheat Straw	15.0	10.0
Total B	30.0	20.0
Total A+B	100	100

* C=Concentrate, R=Roughages

### Body measurement

The whole weight of the animal was weighed on a measuring scale; body height: The animal height was measured as the vertical distance from the thoracic vertebrae to the ground. Body length was measured from the humeri to the aitchbone (*Tuber ischiadicum*). The waist length was collected as the smallest circumference around the animal just behind the foreleg.

### Total feed consumption and feed efficiency measurements

The total feed consumption was determined by calculating the mean of a total feed intake over 2 weeks. The total feed refusal by lambs was calculated by subtracting the total feed given to the lambs and the feed they take in. The feed efficiency was determined by dividing the total feed consumed by the total body weight gain for the experimental period of 2 weeks.

### Biochemical analysis

The biochemical parameters such as crude protein (%), crude fiber (%), and ash (%) were determined according to AOAC [25] and moisture (%) according to ISTA [26]. The analysis of crude protein was conducted using the Kjeldahl Method. Analysis of crude fat was performed using the Soxhlet Apparatus (BUCHI, Switzerland). Analysis of moisture (%) was performed according to ISTA protocols. Analysis of ash (%) was performed according to AOAC protocols [25].

### Statistical analysis

Data were analyzed by least squares analysis of variance. Significant differences between experimental groups with normal distribution and p-value were determined by two-way analysis of variance using Statistical Analysis System software version 6.04 (SAS Inst. Inc., Cary, NC, USA).

## Results

### Effect of different feed additives on the growth rate of young lambs–body weight (kg)

The effect of different feed additives on the body weight of young lambs expressed in kgs is tabulated in Table-2. The pooled data show a significant increase (p < 0.05) in the overall lambs’ body weight. The overall averages of lambs’ body weight displayed the highest of 44.73 ± 3.59 and 44.73 ± 1.46 kg for basal ration supplemented with ammonium chloride and fish meal. Still, the difference between the two groups was insignificant. Urea offered the next higher performance of 39.12 ± 3.37 kg, and the trend continues as algae and humic acid with 36.75 ± 1.76 and 35.44 ± 3.61 kg, respectively.

**Table-2:** Effect of different feed additives on lambs body weight (kg).

Period (every 2 weeks)	Different feed additives added to basal ration					

	Ammonium chloride	Urea	Algae	Fishmeal	Humic Acid	Control
1	28.5 ± 0.0^za^	24.25 ± 0.35^za^	19.5 ± 1.41^za^	26.75 ± 2.47^za^	20.0 ± 6.36^za^	21.0 ± 3.53^za^
2	30.75 ± 1.06^×yz^	25.0 ± 0.71^za^	20.5 ± 1.41^za^	28.75 ± 1.06^za^	20.75 ± 5.30^za^	21.5 ± 4.24^za^
3	33.0 ± 0.70^uvw^	28.25 ± 0.35^za^	23.0 ± 2.82^za^	32.75 ± 1.06^uvw^	24.0 ± 2.12^za^	23.5 ± 4.24^za^
4	36.25 ± 1.76^tuv^	30.0 ± 0.71^wxy^	27.0 ± 2.12^za^	35.75 ± 1.06^tuv^	26.25 ± 1.76^za^	24.5 ± 2.82^za^
5	37.75 ± 1.76^stu^	31.75 ± 1.06^wxy^	28.25 ± 3.88^za^	37.5 ± 1.41^stu^	28.25 ± 0.35^za^	27.5 ± 2.82^za^
6	40.75 ± 2.47^opq^	36.75 ± 2.47^tuv^	34.25 ± 2.47^uvw^	42.25 ± 0.35^klm^	31.0 ± 2.82^wxy^	30.5 ± 1.41^×yz^
7	46.75 ± 2.47^efg^	40.25 ± 1.76^opq^	39.75 ± 1.76^qr^	45.5 ± 2.82^efg^	37.25 ± 1.76^stu^	36.5 ± 0.71^tuv^
8	48.75 ± 3.88^dcd^	40.25 ± 6.01^opq^	41.25 ± 1.76^mno^	47.25 ± 0.35^def^	39.25 ± 2.47^qrs^	36.0 ± 0.70^tuv^
9	51.5 ± 4.24^ab^	45.75 ± 3.88^efg^	43.75 ± 1.76^ijk^	50.5 ± 0^abc^	42.75 ± 3.88^klm^	38.0 ± 2.82^rs^
10	51.5 ± 10.60^ab^	48.25 ± 5.3^bcd^	47.75 ± 0.35^def^	55.0 ± 1.41^a^	44.0 ± 4.24^ghi^	39.5 ± 2.12^qr^
11	56.25 ± 6.01^a^	50 ± 6.36^abc^	49.5 ± 0.70^bcd^	58.25 ± 1.76^a^	45.75 ± 3.88^efg^	40.5 ± 3.53^opq^
12	59.0 ± 5.65^a^	53.25 ± 7.42^a^	51.0 ± 1.41^ab^	60.0 ± 2.82^a^	50.0 ± 5.65^abc^	42.75 ± 4.59^klm^
13	60.75 ± 6.01^a^	54.75 ± 7.42^a^	52.25 ± 1.06^a^	61.25 ± 2.47^a^	51.5 ± 6.36^ab^	43.75 ± 4.59^ijk^
Overall Averages	44.73 ± 3.59^A^	39.12 ± 3.37^B^	36.75 ± 1.76^C^	44.73 ± 1.46^A^	35.44 ± 3.61^C^	32.73 ± 2.93^D^

Values are the means ± the standard deviation. The number of replicates is three. Means within a row and a column with common lower superscripts are not significantly different (p > 0.05). Means with different superscripted upper-case letters are significantly different from each other at (p > 0.05)

### Effect of different feed additives on the growth rate of young lambs–BL (cm)

The reflection of different feed additives to the basal ratio on lamb’s BL is shown in Table-3. The supplementation of feed additives displayed a significant increase in lamb’s BL. The inclusion of ammonium chloride, fish meal, and urea to basic ration performed improved BL of 75.69 ± 1.74, 75.12 ± 1.14 cm, and 74.19 ± 2.67 cm, respectively. The effect of the additives algae and humic acid (70.96 ± 4.62 and 69.39 ± 2.50 cm) was comparatively lesser than control treatments fed with basal ration alone (72.5 ± 2.88 cm).

**Table-3:** Effect of different feed additives on lambs’ body length (cm).

Period (every 2 weeks)	Treatments					

	Ammonium chloride	Urea	Algae	Fishmeal	Humic Acid	Control
1	65.0 ± 1.41^mno^	61.5 ± 0.71^o^	64.5 ± 0.71^no^	66.5 ± 0.70^mno^	59.5 ± 2.12^o^	62.0 ± 5.66^o^
2	67.0 ± 4.24^mno^	63.0 ± 1.41^no^	66.5 ± 0.71^mno^	67.5 ± 0.70^mno^	60 ± 1.41^o^	64.0 ± 1.41^no^
3	68.5 ± 3.3^mno^	70.0 ± 1.41^lmn^	66.0 ± 7.07^mno^	70.5 ± 0.71^lmn^	64.5 ± 2.12^no^	62.0 ± 5.65^o^
4	72.0 ± 1.41^klm^	71.5 ± 0.71^lmn^	66.5 ± 6.36^mno^	72.0 ± 0.0^klm^	65.0 ± 1.41^mno^	65.0 ± 4.24^mno^
5	75.0 ± 1.41^ghi^	73.5 ± 2.12^jkl^	68.0 ± 5.65^mno^	73.5 ± 2.12^jkl^	66.0 ± 1.41^mno^	66.5 ± 4.94^mno^
6	75.0 ± 1.41^ghi^	74.5 ± 2.12^ij^	68.5 ± 3.53^mno^	73.5 ± 2.12^jkl^	66.5 ± 0.71^mno^	69.0 ± 2.82^mno^
7	79.0 ± 5.65^bcd^	72.5 ± 0.71^klm^	68.5 ± 4.94^lmno^	71.5 ± 3.53^lmn^	71.0 ± 1.41^lmn^	67.5 ± 6.36^mno^
8	75.0 ± 1.41^ghi^	77.5 ± 0.71^def^	70.0 ± 4.24^lmn^	75.0 ± 0.0^jhi^	68.0 ± 0.0^mno^	76.0 ± 2.83^jh^
9	76.5 ± 0.71^efg^	76.0 ± 5.66^gh^	71.5 ± 4.95^lmn^	76.5 ± 0.71^efg^	70.0 ± 0.0^lmn^	79.5 ± 0.71^abc^
10	79.5 ± 0.71^abc^	78.0 ± 5.66^cde^	74.5 ± 6.36^ij^	79.5 ± 0.71^abc^	74.0 ± 2.83^ij^	80.5 ± 0.71^ab^
11	81.5 ± 0.71^a^	80.5 ± 4.95^ab^	77.0 ± 5.66^def^	81.0 ± 1.41^a^	77.0 ± 4.24^def^	81.5 ± 0.71^a^
12	84.0 ± 0^a^	82.5 ± 4.95^a^	79.0 ± 5.66^bcd^	84.0 ± 1.41^a^	79.5 ± 6.36^abc^	83.5 ± 0.71^a^
13	86.0 ± 0^a^	83.5 ± 3.54^a^	82.0 ± 4.24^a^	85.5 ± 0.71^a^	81.0 ± 8.48^a^	85.5 ± 0.71^a^
Overall Averages	75.69 ± 1.74^A^	74.19 ± 2.67^AB^	70.96 ± 4.62^CD^	75.12 ± 1.14^A^	69.39 ± 2.50^D^	72.5 ± 2.88^BC^

Values are the means ± the standard deviation. The number of replicates is three. Means within a row and a column with common lower superscripts are not significantly different (p > 0.05). Means with different superscripted upper-case letters are significantly different from each other at (p > 0.05)

### Effect of different feed additives on the growth rate of young lambs–body height (cm)

The effect of various feed additives on the body height of young lambs, expressed in cms, is tabulated in Table-4. The results showed that adding various feed additives to the basal ration could significantly improve significantly (p < 0.05) lambs’ body height. The additives, fish meal, and ammonium chloride recorded the highest body heights of 77 ± 1.31 and 77.69 ± 1.2 cm, followed by urea, recording 75.42 ± 1.69 cm. The body height of the feed additives, humic acid, and algae showed a non-significant increase and recorded the lowest body height of 72.19 ± 2.12 and 71.07 ± 2.72 cm, which is lower than the control without feed additives, recording 74.04 ± 2.12 cm.

**Table-4:** Effect of different feed additives on lambs’ body height (cm).

Period (every 2 weeks)	Different feed additives added to basal ration					

	Ammonium chloride	Urea	Algae	Fishmeal	Humic Acid	Control
1	66.0 ± 1.41^uv^	65.5 ± 2.12^uv^	64.0 ± 0.0^uv^	68.0 ± 1.41^tuv^	63.5 ± 0.71^v^	64.0 ± 4.24^uv^
2	76.5 ± 2.12^jkl^	67.5 ± 2.12^tuv^	64.0 ± 0.0^uv^	68.0 ± 2.82^tuv^	64.0 ± 0.0^uv^	63.5 ± 3.53^v^
3	72.5 ± 0.71^pqr^	70.0 ± 0.0^qrs^	67.5 ± 6.36^tuv^	71.5 ± 0.71^qrs^	64.0 ± 1.41^uv^	67.0 ± 1.41^tuv^
4	72.5 ± 0.71^pqr^	71.0 ± 0.0^qrs^	67.5 ± 4.94^tuv^	71.5 ± 0.71^qrs^	66.5 ± 2.12^uv^	67.0 ± 1.41^tuv^
5	75.0 ± 2.82^lmn^	71.5 ± 0.71^qrs^	69.5 ± 2.12^rst^	73.5 ± 0.71^opq^	68.0 ± 0.0^tuv^	69.5 ± 3.53^rst^
6	76.0 ± 1.41^jkl^	73.5 ± 2.12^opq^	70.0 ± 2.82^qrs^	74.5 ± 0.71^nop^	69.0 ± 0.0^rst^	69.0 ± 2.82^rst^
7	77.5 ± 0.71^hij^	75.0 ± 4.24^lmn^	70.0 ± 4.24^qrs^	75.0 ± 4.24^lmn^	73.0 ± 2.82^opq^	71.5 ± 0.71^qrs^
8	76.0 ± 2.83^jkl^	74.5 ± 4.95^nop^	70 ± 2.83^qrs^	78.0 ± 0.0^hij^	71.0 ± 2.83^qrs^	72.0 ± 0.0^pqr^
9	80.0 ± 0.0d^ef^	78.5 ± 2.12^fgh^	71.5 ± 3.53^qrs^	79.0 ± 0.0^def^	75.0 ± 1.41^lmn^	81.0 ± 2.82^bcd^
10	82.0 ± 1.41^abc^	80.5 ± 0.71^bcd^	74.0 ± 4.24^nop^	83.0 ± 1.41^ab^	78.5 ± 2.12^fgh^	83.0 ± 1.41^ab^
11	84.0 ± 1.41^a^	82.5 ± 0.71^abc^	77.5 ± 2.12^hij^	84.5 ± 0.71^a^	80.5 ± 3.54^bcd^	84.0 ± 1.41^a^
12	85.0 ± 0.0^a^	84.5 ± 0.71^a^	78.5 ± 0.71^fgh^	87.0 ± 1.41^a^	82.0 ± 4.24^abc^	84.5 ± 2.12^a^
13	87.0 ± 0.0^a^	86.0 ± 1.41^a^	80.0 ± 1.41^def^	87.5 ± 2.12^a^	83.5 ± 6.36^a^	86.5 ± 2.12^a^
Overall Averages	77.69 ± 1.2^A^	75.42 ± 1.69^B^	71.07 ± 2.72^D^	77 ± 1.31^A^	72.19 ± 2.12^D^	74.04 ± 2.12^C^

Values are the means ± the standard deviation. The number of replicates is three. Means within a row and a column with common lower superscripts are not significantly different (p > 0.05). Means with different superscripted upper-case letters are significantly different from each other at (p > 0.05)

### Effect of different feed additives on the growth rate of young lambs–waist length (cm)

The effect of various feed additives on the body waist of young lambs (cm) is enumerated in Table-5. The results indicated that the selected feed additives used in this study could increase the waist length of young lambs. A significant increase in the overall averages of lambs’ waist length, as compared to the control group, was recorded. The waist length augmented significantly (p < 0.05) after feeding the young lambs with rations supplemented with the following feed additives; ammonium chloride or urea, recording 85.69 ± 4.46 and 85.46 ± 0.76 cm. However, the increase in lambs’ body heart girth when fed with ration supplemented with ammonium chloride and fishmeal was insignificant when comparing these groups together. The basic feed ration supplemented with urea showed the next best performance of 81.07 ± 1.31 cm, followed by algae and humic acid, showing 76.27 ± 2.77 and 75.85 ± 4.13 cm, respectively.

**Table-5:** Effect of different feed additives on lambs’ body waist (cm).

Period (every 2 weeks)	Treatments					

	Ammonium Chloride	Urea	Algae	Fishmeal	Humic acid	Control
1	71.5 ± 2.12^uvw^	70.5 ± 0.71^uvw^	64 ± 2.82^w^	73 ± 0.0^stu^	65.5 ± 4.94^w^	66.0 ± 7.07^w^
2	76.5 ± 2.12^qrs^	73 ± 1.41^stu^	64 ± 2.82^w^	71 ± 1.41^uvw^	66 ± 4.24^w^	67.0 ± 8.48^vw^
3	76 ± 4.24^qrs^	73.5 ± 2.12^stu^	68.5 ± 6.36^uvw^	74.5 ± 0.71^stu^	66.5 ± 3.53^w^	67.5 ± 4.94^uvw^
4	78.5 ± 6.36^opq^	74.5 ± 2.12^stu^	70.5 ± 7.77^uvw^	76.5 ± 0.71^qrs^	69 ± 1.41^uvw^	67.0 ± 4.24^vw^
5	81 ± 2.82^klm^	78.5 ± 2.12^opq^	73 ± 4.24^stu^	80 ± 0.0^mno^	72 ± 1.41^uvw^	70.5 ± 4.94^uvw^
6	84 ± 4.24^efg^	82.5 ± 0.71^ijk^	77.5 ± 2.12^qrs^	82 ± 1.41^ijk^	74 ± 1.41^stu^	71.5 ± 4.94^uvw^
7	89 ± 4.24^bcd^	84 ± 1.41^efg^	76 ± 1.41^qrs^	84 ± 0.0^efg^	78 ± 1.41^opq^	80.0 ± 1.41^mno^
8	87.5 ± 4.95^bcde^	83.5 ± 0.71^ghi^	76.5 ± 10.61^qrs^	86 ± 1.41^cde^	76 ± 2.83^qrs^	75.0 ± 7.07^stu^
9	91.0 ± 2.83^abc^	85.5 ± 2.12^cdef^	86.5 ± 3.54^cde^	88 ± 0^bcd^	81 ± 1.41^klm^	82.5 ± 2.12^ijk^
10	93.0 ± 7.07^ab^	83.5 ± 0.71^ghi^	88 ± 1.41^bcd^	97.5 ± 0.71^a^	82 ± 1.41^ijk^	83.5 ± 2.12^ghi^
11	93.5 ± 6.36^ab^	86 ± 1.41^cde^	88.5 ± 0.71^bcd^	98 ± 1.41^a^	85 ± 2.83^cdef^	82.5 ± 0.71^ijk^
12	95.5 ± 4.95^ab^	88.5 ± 0.71^bcd^	90.5 ± 0.71^bcd^	99.5 ± 0.71^a^	87.5 ± 3.54^bcde^	85.5 ± 2.12^cdef^
13	97 ± 5.66^a^	90.5 ± 0.71^bcd^	93 ± 1.41^ab^	101 ± 1.41^a^	89 ± 5.66^bcd^	87.5 ± 3.54^bcde^
Overall Averages	85.69 ± 4.46^A^	81.07 ± 1.31^B^	78.19 ± 3.54^C^	85.46 ± 0.76^A^	76.27 ± 2.77^D^	75.85 ± 4.13^D^

Values are the means ± the standard deviation. The number of replicates is three. Means within a row and a column with common lower superscripts are not significantly different (p > 0.05). Means with different superscripted upper-case letters are significantly different from each other at (p > 0.05). Age lambs 4–4.5 months’ data were collected every 2 weeks, and the total period of collecting data is 26 weeks

### Effect of different feed additives on the total feed consumption of young lambs (kg/2 weeks)

The total feed consumption of the young lambs after intake of different feed additives in the diet is shown in Table-6. The total feed consumption of young lambs fed with a ration supplemented with algae, fish meal, and urea was significantly higher, recording 13.90 ± 4.09, 13.89 ± 4.37, and 13.89 ± 4.27 kg, respectively. Feed supplemented with ammonium chloride recorded 13.89 ± 4.27 kg consumption, which is on par with the control recording of 13.75 ± 23.01 kg. Humic acid as a feed additive recorded the lowest performance of 13.42 ± 66.43 kg.

**Table-6:** Effect of different feed additives on total feed consumption of young lambs (kg/2 weeks).

Period (every 2 weeks)	Treatments					

	Ammonium Chloride	Urea	Algae	Fishmeal	Humic acid	Control
1	-	-	-	-	-	-
2	13.17 ± 0.35^cde^	12.99 ± 55.86^de^	13.24 ± 9.54^cde^	13.14 ± 37.61^cde^	12.96 ± 33.58^de^	12.79 ± 36.62^de^
3	13.89 ± 13.43^abc^	13.85 ± 13.43^abc^	14.00 ± 0.00^a^	13.96 ± 1.41^a^	13.58 ± 34.64^cde^	13.97 ± 3.54^a^
4	13.92 ± 3.53^ab^	14.00 ± 0.00^a^	13.83 ± 24.04^bcd^	13.88 ± 5.65^abc^	13.17 ± 86.26^cde^	13.95 ± 56.57^a^
5	13.90 ± 14.14^ab^	13.70 ± 8.48^cde^	13.74 ± 15.55^cde^	14.00 ± 0.00^a^	13.52 ± 57.98^cde^	13.91 ± 12.21^ab^
6	13.86 ± 19.79^bcd^	13.94 ± 2.82^ab^	14.00 ± 0.00^a^	14.00 ± 0.00^a^	13.26 ± 103.94^cde^	13.93 ± 9.89^ab^
7	14.00 ± 0.00^a^	14.00 ± 0.00^a^	14.00 ± 0.00^a^	14.00 ± 0.00^a^	13.22 ± 109.60^cde^	14.00 ± 0.00^a^
8	14.00 ± 0.00^a^	14.00 ± 0.00^a^	14.00 ± 0.00^a^	14.00 ± 0.00^a^	3.76 ± 33.23^cde^	14.00 ± 0.00^a^
9	14.00 ± 0.00^a^	14.00 ± 0.00^a^	14.00 ± 0.00^a^	14.00 ± 0.00^a^	13.60 ± 56.56^cde^	14.00 ± 0.00^a^
10	14.00 ± 0.00^a^	12.86 ± 160.51^de^	14.00 ± 0.00^a^	14.00 ± 0.00^a^	12.72 ± 180.31^e^	14.00 ± 0.00^a^
11	14.00 ± 0.00^a^	13.13 ± 122.32^cde^	14.00 ± 0.00^a^	14.00 ± 0.00^a^	13.50 ± 70.71^cde^	12.81 ± 167.5^de^
12	14.00 ± 0.00^a^	14.00 ± 0.00^a^	14.00 ± 0.00^a^	13.90 ± 13.43^ab^	13.90 ± 14.14^ab^	13.82 ± 25.45^bcd^
13	14.00 ± 0.00^a^	14.00 ± 0.00^b^	14.00 ± 0.00^a^	13.87 ± 18.38^abc^	13.88 ± 16.26^abc^	13.86 ± 19.0^bcd^
Overall Averages	13.89 ± 4.27^A^	13.70 ± 30.28^A^	13.90 ± 4.09^A^	13.89 ± 4.37^A^	13.42 ± 66.43^B^	13.75 ± 23.01^A^

Values are the means ± the standard deviation. The number of replicates is three. Means within a row and a column with common lower superscripts are not significantly different (p > 0.05). Means with different superscripted upper-case letters are significantly different from each other at (p > 0.05)

### Effect of different feed additives on the feed efficiency of young lambs (g/2 weeks)

Feed efficiency, that is, the conversion of the animal feed into the desired output of meat by lamb body metabolism, is calculated and presented in Table-7. The results clearly showed that adding various feed additives used in this study could increase the feed efficiency of young lambs. The basic feed rations supplemented with fish meal and ammonium chloride expressed higher feed efficiencies of 3.32 ± 0.09 and 3.309 ± 0.27 g, followed by urea representing 2.95 ± 0.31 g. Algae and humic acid expressed the lowest feed efficiency of 2.74 ± 0.18 and 2.73 ± 0.23 g. The control lambs fed without feed additives offered a feed efficiency of only 2.47 ± 0.14 g, which is very meager compared with additives-fed lambs.

**Table-7:** Effect of different feed additives on feed efficiency of young lambs (g/2 wks).

Period (every 2 weeks)	Treatments					

	Ammonium Chloride	Urea	Algae	Fishmeal	Humic acid	Control (no additives)
1	-	-	-	-	-	-
2	2.33 ± 0.09	1.93 ± 0.18	1.55 ± 0.14	2.19 ± 0.00	1.60 ± 0.43	1.68 ± 0.32
3	2.38 ± 0.09	2.04 ± 0.00	1.64 ± 0.23	2.35 ± 0.09	1.77 ± 0.11	1.98 ± 0.35
4	2.60 ± 0.15	2.14 ± 0.06	1.95 ± 0.13	2.58 ± 0.10	1.99 ± 0.02	1.76 ± 0.23
5	2.72 ± 0.11	2.32 ± 0.11	2.06 ± 0.36	2.68 ± 0.12	2.09 ± 0.16	1.98 ± 0.26
6	2.94 ± 0.15	2.64 ± 0.20	2.45 ± 0.21	3.02 ± 0.30	2.34 ± 0.01	2.19 ± 0.14
7	3.34 ± 0.20	2.88 ± 0.15	2.84 ± 0.15	3.25 ± 0.24	2.82 ± 0.17	2.57 ± 0.06
8	3.48 ± 0.33	2.88 ± 0.51	2.95 ± 0.15	3.38 ± 0.03	2.85 ± 0.31	2.57 ± 0.06
9	3.68 ± 0.33	3.27 ± 0.51	3.13 ± 0.15	3.61 ± 0.03	3.14 ± 0.31	2.71 ± 0.06
10	3.68 ± 0.33	3.75 ± 0.51	3.41 ± 0.15	3.93 ± 0.03	3.47 ± 0.31	2.82 ± 0.06
11	4.02 ± 0.33	3.80 ± 0.51	3.54 ± 0.15	4.16 ± 0.03	3.39 ± 0.31	3.17 ± 0.06
12	4.21 ± 0.33	3.80 ± 0.51	3.64 ± 0.15	4.31 ± 0.03	3.60 ± 0.31	3.09 ± 0.06
13	4.34 ± 0.33	3.91 ± 0.51	3.73 ± 0.15	4.42 ± 0.03	3.71 ± 0.31	3.15 ± 0.06
Overall averages	3.309 ± 0.27^A^	2.95 ± 0.31^B^	2.74 ± 0.18^C^	3.32 ± 0.09^A^	2.73 ± 0.23^C^	2.47 ± 0.14^D^

Values are the means ± the standard deviation. The number of replicates is three. Means within a row and a column with common lower superscripts are not significantly different (p > 0.05). Means with different superscripted upper case letters are significantly different from each other at (p < 0.05)

### Biochemical analysis of feed additives

The biochemical parameters of the feed samples are shown in Table-8. The crude protein content was highest in a diet supplemented with fish meal (28.56%), followed by urea (22.02%) and ammonium chloride (18.64%). The crude protein content of the basal lamb ration and algae was almost the same (12%), and humic acid was reported the least (8.8%). The crude fiber content was maximum in fishmeal (13.6%t). Feed additives ammonium chloride, algae, and urea reported similar results of 4.16, 3.97, and 3.79%, which were on par with the basal feed ration of 3.73%. The crude fiber content was lowest in humic acid. The ash content was the highest and lowest recorded in fish meal and humic acid at 4.29% and 1.20%. The other additives, algae, ammonium chloride, and urea (2.29, 2.21, and 2.04), were on par with the basal feed ration (2.64%). The moisture content was highest in ammonium chloride and urea (90%), followed by algae (89%), least in humic acid (7.44%), and fish meal (6.40%).

**Table-8:** Biochemical analysis of experimental rations and feed additives.

Treatments	Ash	Crude fiber	Crude protein	Moisture
Ammonium chloride	2.21 ± 0.04	4.16 ± 0.18	18.64 ± 0.09	90.93 ± 0.03
Urea	2.04 ± 0.06	3.79 ± 0.08	22.02 ± 0.00	90.23 ± 0.19
Algae	2.29 ± 0.05	3.97 ± 0.32	12.67 ± 0.02	89.94 ± 0.16
Fishmeal	4.29 ± 0.02	13.6 ± 0.05	28.56 ± 0.13	6.40 ± 0.55
Humic acid	1.20 ± 0.03	0.28 ± 0.01	8.80 ± 0.19	7.44 ± 0.19
Lamb’s ration (80C: 20R)	2.64 ± 0.07	3.73 ± 0.31	12.45 ± 0.12	9.37 ± 0.24

## Discussion

The investigations of lambs with five rations, developed using five feed additives, including; ammonium chloride, urea, algae, fishmeal, and humic acid, exhibited significant amplification in lamb’s performance compared to the control without feed additives. Of the tested additives, ammonium chloride and fish meal were efficient in amplifying the physical performance of animals, such as body weight, length, height, and waist length, and improved the total feed consumption and feed efficiency of the examined lambs. An increase in body weight is an essential criterion for lambs reared for meat purposes. The data revealed that supplementing the ration with diverse feed additives could be very effective in increasing lambs’ body weight and could be recommended to farmers for the fattening process of young lambs. Lambs fed with rations, including ammonium chloride and fish meal additives, showed a vast increment in body weight (44.3 kg) compared to lambs fed with basal rations without feed additives (32.73 kg). Additives, ammonium chloride, and fishmeal are strongly recommended as they could significantly increase the body weight of lambs. The elevated BLs of 75.69 ± 1.74, 75.12 ± 1.14 cm, body height of 77 ± 1.31 and 77.69 ± 1.2 cm, and body waist level of 85.69 ± 4.46 and 85.46 ± 0.76 cm, respectively, for ammonium chloride and urea was recorded, which was highest among all additives investigated. The total feed consumption also recorded the highest of 13.89 kg for ammonium chloride and fish meal supplementation. The basic feed rations supplemented with fish meal and ammonium chloride expressed higher feed efficiency of 3.32 ± 0.09 and 3.309 ± 0.27 g.

In general, ammonium chloride is applied as an acidity regulator in the feed of ruminants. Supplementing the ration with ammonium chloride could acidify the urine, which will help prevent the buildup of calculi or stones, which is an essential metabolic disease in sheep, where the formation of stones in the urinary tract will prevent urination [27]. The addition of ammonium chloride at a rate of 0.35% in the complete ration will decrease the urine pH from 6.9–5.9. In this case, it could be considered as an acidity regulator of feed for small ruminants [28]. Ammonium chloride supplementation keeps the lambs healthy to withstand these metabolic diseases and increases their physical performance, especially by increasing their body weight. This finding is in accordance with that of Mary *et al*. [29], who expressed the potential of ammonium chloride in enhancing the body weight of goats. Moreover, adding ammonium chloride could lower the blood pH, which will help in the metabolism of calcium reserves in the bone. This additional calcium will help protect the animal against milk fever [30]. *In vitro* studies have corroborated that ammonium chloride is an excellent nitrogen source for rumen microbial cell growth and starch digestion. Ammonium chloride contains higher crude protein (18.64%), fiber (4.16%), and moisture content (90.93%), which makes it highly nutritious. When ammonium chloride was added to mixed lamb rations, feed efficiency was increased over a diet containing cotton-seed meal as the supplementary nitrogen source [31, 32]. When the lambs are healthy and unaffected by metabolic diseases due to ammonium chloride addition, the body weight increases and, ultimately, the body height, weight, waist length, etc. As the body is healthy, they consume feed effectively and record good feed efficiency. The ammonia in the animal body is detoxified by being metabolized to urea through the urea cycle in the liver; therefore, no toxic effects of the ions will be shown in the animal body [33, 34]. It is obvious from this study that ammonium chloride as a feed additive augmented the body weight, length, height, waist, and feeding efficiency of lambs, which is in concordance with that of Gabriele *et al*. [35].

The efficacy of fish meals in augmented growth and feed consumption and efficiency capabilities follows previous findings [36–39]. Fish is an excellent source of high protein for ruminants, which slowly degrades in the rumen with an excellent amino acid profile [40]. Fish meal is a powdered dried fish formulation with water and oil removed. Fishmeal contains the highest level of crude proteins (28.56%), crude fiber (13.6%), and ash (4.29%) in dry matter (DM). Because of its high protein content, fish meal helps in boosting the immune system, increases growth rate, reduces the worm burden, and enhances embryo survival. In addition, fishmeal is rich in essential fatty acids. Fishmeal contains digestible, un-degradable protein that passes through the rumen [41, 42]. The degradation of fishmeal protein has been reported to improve the fiber digestion and productivity of the animals. In addition, omega-3 fatty acids in fishmeal could enhance fatty acid uptake and improve fertility, the growth rate of young lambs, and immunity. A recommended rate of 35 g/1 kg is suggested for its usage as a feed additive for animals [43]. The current research findings showed that feeding young lambs with rations supplemented with fishmeal powder was effective in increasing the body weight of young lambs, which is inconsistent with the previous findings [44–46].

Urea is yet another feed additive that portrayed the best effects on lamb’s performance physically with live weight and growth performance and regarding feed efficiency. The basal ration supplemented with urea in this study increased the body weight to 39.12 kg significantly. The growth parameters, BL, height, and waist length recorded an elevated level of 74.19 ± 2.67, 75.42 ± 1.69 cm, and 81.07 ± 1.31 cm due to urea supplement with the basal ration. The results are consistent with Mahdi *et al*. [47], stating that urea can be substituted instead of soybean meal with N-carbamylglutamate addition without negative effects on animals, and increasing feed efficiency, increasing daily gains and total weights, improving the productive features of Awassi lambs. The universal, non-protein nitrogen source used in ruminant feeding is urea, which is an inexpensive nitrogenous compound. It could substitute some degradable protein in the animal ration, which could help in lambs fattening [48, 49]. The addition of urea to livestock ration containing barley grain was reported to change the rate of ruminal fermentation, quantities of some ruminal bacterial populations, and activity of some enzymes [50, 51]. The previous report suggest the use of urea in the livestock ration at a rate of 1% of the total ration or approximately 3% of the concentrate mixture, especially in the case of feeding the animals with rations, containing low percentages of roughage [52]. The total digestible nutrient content of the ration affects urea usage; diet with high grain results in good urea usage, and high forage results in lowered usage of urea [53]. It was evident in this study that the inclusion of urea at the basal ration elevated the physical performance of lambs, with is in coherence with numerous previous studies [54–61].

Algae is another investigated feed additive that benefits lamb performance in total feed consumption. In this study, the basal ration supplemented with algae as a feed additive enhanced total feed consumption to 13.90 kg, which is the highest among all examined feed additives. This finding is inconsistent with several previous studies stating that adding seaweed to livestock ration is believed to improve the feed consumption rate and wool quality [62]. The feed efficiency of lambs witnessed 2.74 g, which is next to the lowest of the investigated additives. Algae are rich in many minerals that most animals require for their basic bodily functions, including phosphorus, zinc, magnesium, and iron [63]. Algae for animal feed can help improve an animal’s intestinal health and activate the animal’s immune system. They contain bioactive compounds known to have antioxidant, anti-inflammatory, and anti-viral qualities, generally pronounced as prebiotics, which are functional compounds for gut health [64–67]. Laboratory analyses of algae showed more than 60 minerals such as; calcium (390–1005 mg/100 g), Mn (1.32 mg/100 g), potassium (3,184–11,579 mg/100 g), sodium (3627–7064 mg/100 g), zinc (1.74–7.14 mg/100 g), manganese (565–1,181 mg/100 g), iron (3.29–10.3 mg/100 g), and selenium [68]. In addition, algae contain protein, fat, carbohydrates, plant growth hormones, and amino acids such as lysine, histidine, and proline. Because of the presence of these nutrients, algae are essential for the growth of beneficial microorganisms found in the gastrointestinal tract [69]. Thus, the addition of algae to the livestock diet will act as an alternative to antibiotic growth promoters [70]. The previous research stated that algae as feed additives increase milk production in cattle flocks when added to ration and could improve animals’ conception rate and reduce the mastitis rate, due to its content of selenium and tocopherol [71]. In this investigation, the addition of algae to the basal ration of young lambs was found to improve the body weight and growth performance of young lambs. Still, the performance was lower than that of ammonium chloride, fish meal, and algae. Algae showed the least performance next to humic acid from the lowest. The lamb body weight was 36.75 kg, BL witnessed 70.96 cm, body height 72.19 kg, and body waist level of 76.27 cm. The results agree with previous findings stating that algae as feed additives improve the performance and immunity of livestock due to its content of probiotic compounds, which act as; antibacterial, anti-viral, anti-inflammatory, and antioxidant [72].

Although the efficacy of humic acid is least compared with other additives investigated, humic acid did possess qualities for the physical performance of lambs. It supplements as feed additives with the investigated animals witnessing 35.44 ± 3.61 kg body weight, 69.39 cm BL, 71.07 cm body height, and 75.85 cm body waist level. The findings agree with Islam *et al*. [8], stating that adding humic acid could help increase the body weight of animals without increasing the amount of feed used. In contrast, the effect of the addition of humic acid to the basal ration of lambs did not have any significant effect on the immune status of these animals. The most common form of organic carbon in the environment is from humic substances- in the form of decomposed plant and animal matter. Humic acid contains 4% nitrogen; thus, it could be added to livestock ration, as previously mentioned, at a rate of 2.5 g/1 kg DM [7]. The calves born to cows fed with humate had 13.4% augmented weight within 4 months in a study by Pisarikova *et al*. [73]. It was reported previously that humic acids have a significant effect on the growth performance of animals and help boost the immunity of different animal species [74], which is consistent with our present results as supplementing the ration with humic acid increased lambs’ body weight and growth performance compared with control without feed additives. Humic acid’s capability in increased physical performance and feed efficiency was lower than other investigated additives, but it has a significant difference compared to the control.

## Conclusion

This study concluded an affirmative escalation in the physical performance of lambs through the supplementation of feed additives, especially ammonium chloride and fishmeal. The physical growth parameters of length, height, and waist length of lambs’ bodies increased significantly when supplemented with ammonium chloride and fishmeal. Urea exhibited a neutral effect, while algae and humic acid disclosed minimal performance in the body physique of lambs. The total feed consumption of young lambs fed with algae, fish meal, and urea as feed additives portrayed the best, with ammonium chloride having a neutral effect, while humic acid was the least. The feed efficiency of young lambs was elevated with fish meal and ammonium chloride, followed by urea. The study was intended to manufacture assorted feed mixtures by including the best-feed additives appropriate for sheep in Kuwait. Consequently, the supplementation of additives in the order of ammonium chloride, fishmeal, urea, algae, and humic acid in animal feed production to advance sheep growth performance and immunity for sustainable sheep production in Kuwait are recommended.

## Authors’ Contributions

HB: Conceptualized, designed, and supervised the experimental work and reviewed the manuscript. FK: Conducted the study, statistically analyzed and interpreted data, and drafted and edited the manuscript. All authors have read and approved the final manuscript.
